# Three-Dimensional Reconstruction of Road Structural Defects Using GPR Investigation and Back-Projection Algorithm

**DOI:** 10.3390/s25010162

**Published:** 2024-12-30

**Authors:** Lutai Wang, Zhen Liu, Xingyu Gu, Danyu Wang

**Affiliations:** 1 Department of Roadway Engineering, School of Transportation, Southeast University, Nanjing 211189, China; wlt@seu.edu.cn (L.W.); 220173387@seu.edu.cn (D.W.); 2 Zhejiang Rail Transit Construction Management Group Limited, Hangzhou 311200, China; 3 Department Civil and Environmental Engineering Department, Pennsylvania State University, University Park, State College, PA 16801, USA

**Keywords:** ground-penetrating radar, non-destructive testing, three-dimensional reconstruction, BP imaging

## Abstract

Ground-Penetrating Radar (GPR) has demonstrated significant advantages in the non-destructive detection of road structural defects due to its speed, safety, and efficiency. This paper proposes a three-dimensional (3D) reconstruction method for GPR images, integrating the back-projection (BP) imaging algorithm to accurately determine the size, location, and other parameters of road structural defects. Initially, GPR detection images were preprocessed, including direct wave removal and wavelet denoising, followed by the application of the BP algorithm to effectively restore the defect’s location and size. Subsequently, a 3D data set was constructed through interpolation, and the effective reflection data were extracted by using a clustering algorithm. This algorithm distinguished the effective reflection data from the background data by determining the distance threshold between the data points. The 3D imaging of the defect was then performed in MATLAB. The proposed method was validated using both gprMax simulations and laboratory test models. The experimental results indicate that the correlation between the reconstructed and actual defects was approximately 0.67, demonstrating the method’s efficacy in accurately achieving the 3D reconstruction of road structural defects.

## 1. Introduction

The digitization of road infrastructure is not only the future development direction of the transportation industry but also one of the critical processes that need continuous attention and solutions to achieve long-term, high-quality road development and improve road service resilience [1,2]. At present, the road industry is entering the maintenance stage from the large-scale construction stage. How to efficiently complete road detection without damage has become a hot research issue [3].

Due to traffic, environmental, and other factors, roads are prone to defects such as cracks, cavities, and pits. These defects can generally be divided into road surface defects and internal structural defects [4]. Ruts, pits, and other surface defects are easy to detect and identify among them. However, because hidden cracks, cavities, and other structural defects are located in the interior of the road, it is not easy to detect them. If not found and treated in time, they may extend to the surface, causing more severe damage, such as pavement collapse. Traditional detection methods for road structural defects, such as drilling coring and bending detection, are no longer suitable for large-scale road maintenance due to the disadvantages of destructiveness, high cost, and incomplete detection.

Ground-Penetrating Radar (GPR) technology has been gradually used in road detection since the 1980s. Because of its nondestructive, fast detection speed and high degree of automation, it has developed relatively rapidly [5,6,7]. It transmits electromagnetic waves to the interior of the road structure. These electromagnetic waves will have different degrees of reflection and refraction when they meet the two media with different dielectric constants during propagation, which can be used for the assessment.

At present, through the efforts of many researchers all over the world, GPR technology has made significant progress in various aspects, such as pavement thickness measurement [8,9,10,11], underground pipeline detection [12,13,14], road moisture content and porosity detection [15,16,17], and road structural defects detection [4,18,19,20], and has been gradually applied to practical projects. Among them, GPR-based 3D reconstruction technology can quickly and intuitively acquire the location, scale, and other important information of road structural defects, which has excellent development potential [21].

Guo et al. [22,23] used the Finite-Difference Time-Domain (FDTD) principle to build the dielectric constant model of asphalt mixture and analyzed the GPR image characteristics of crack defects on this basis, that is, the reflected image presents a double-curve shape, and the amplitude intensity increases with the increase in the crack width. Rasol et al. [24,25] used the results of an indoor road structural defects model test and numerical simulations to analyze GPR images of cracks and cavities, where they demonstrated the influence of the defect size on waveform images. However, this research only made a simple two-dimensional determination of defects from the features of simulated or detected GPR images. They cannot obtain more information, such as the form, scale, and depth of defects. Using the F-K migration algorithm, Zhang et al. [26] performed clutter removal and scattering strength repair for GPR images of structural defects on a bridge deck, effectively restoring the initial position of steel bars and holes on the bridge deck, but the error was relatively large. Mauricio Pereira et al. [27] proposed a process of generating 3D models based on the original data from 3D GPR and successfully applied it to underground targets of three different sizes and shapes. Zhu et al. [28] used 3D GPR to detect the size and distribution of tree roots, and realized 3D visualization of underground roots by automatically setting amplitude thresholds. Deng et al. [29] studied the 3D imaging technology of tunnel slump GPR based on a half-space scanning measurement mode. Hu et al. [30] used 3D GPR to detect the leakage of urban municipal pipelines and achieved good results.

However, in actual field detection, the type, size, and shape of road structural defects vary, which is critical for the stress intensity factor [31,32] and the stability of the road. The composition of the underground medium is complex, including soil, rock, asphalt, concrete, and water, and the external electromagnetic noise will also cause certain interference. Moreover, the map processing and analysis of GPR detection data are complicated. All these factors restrict the development of 3D reconstruction technology for road structural defects based on GPR. To solve this problem, a 3D reconstruction method for the GPR data of road structural defects combined with a BP imaging algorithm is proposed in this paper, and the method was verified by 3D defect models simulated by gprMax 3.0 software and laboratory test models. This method can effectively filter the interference of noise, as well as detect and reconstruct the road structural defects more accurately.

## 2. Methodology

### 2.1. Three-Dimensional Reconstruction Method for Road Structural Defects

**Figure 1:**
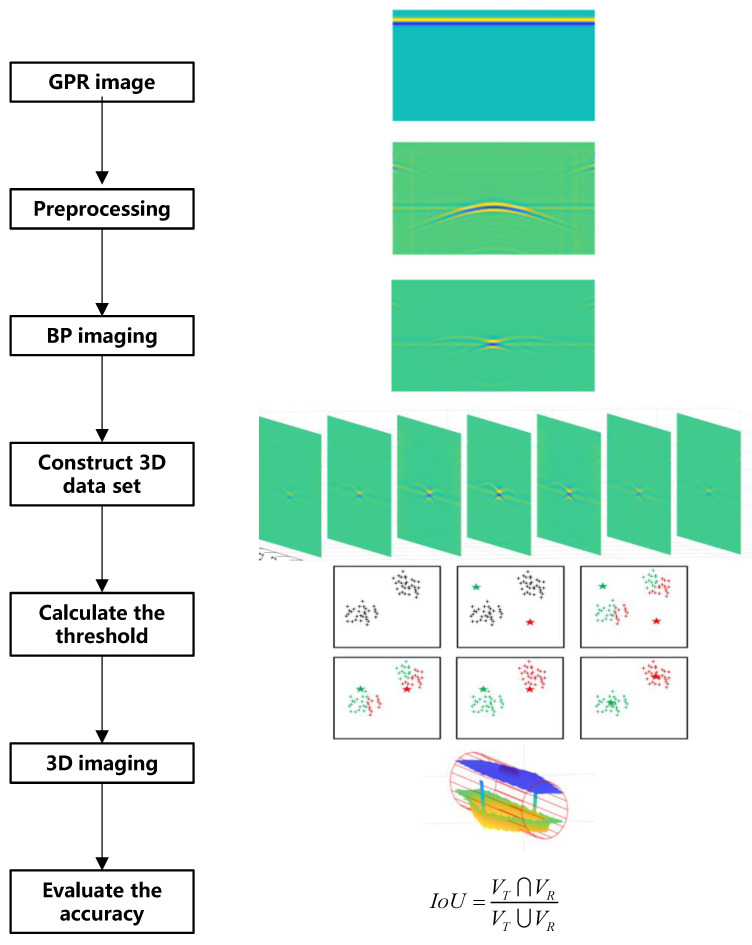
Three-dimensional reconstruction process for road structural defects.

Based on simulated and measured GPR detection data, this study adopted the following steps for the 3D reconstruction of road structural defects. The flowchart is shown in Figure 1.

(1) Preprocessing: direct wave removal and wavelet denoising were carried out on the GPR detection data of each section to remove these noises to the maximum extent, improve the signal-to-noise ratio of the image, and retain effective target reflection information [33].
(2) BP imaging: A BP (back-projection) imaging algorithm was used to process the pre-processed GPR data to realize the return of the reflected wave and the convergence of the diffraction wave, and then effectively restore the location and size of the defect to ensure the accuracy of the 3D reconstruction.
(3) Construct a 3D data set: through interpolation, the collected data of each section were combined into a 3D data set.
(4) Calculate the threshold: the K-means-AHC algorithm was used to calculate the threshold of distinguishing background data and effective reflection data, and all the former were set to 0, while only the latter were retained.
(5) Three-dimensional imaging: the effective reflection data’s upper and lower surfaces were extracted and processed in MATLAB 2022 [34] to realize the 3D imaging.
(6) Evaluation accuracy: according to the location and size of the reconstructed disease and its actual location and size, the *IoU* (Intersection Over Union) was calculated to evaluate the accuracy of the 3D reconstruction.

### 2.2. Principle of GPR Detection

**Figure 2:**
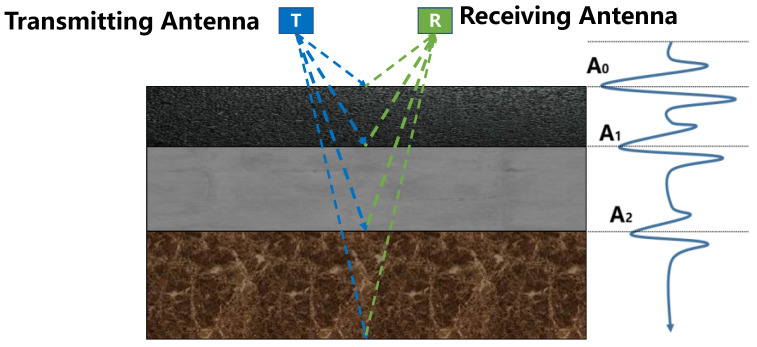
Principle of GPR detection.

GPR transmits high-frequency electromagnetic wave signals to underground media through transmitting antennas. When the electromagnetic wave propagates to the underground dielectric interface with different dielectric constants, corresponding reflections will be generated. Receiving antennas capture the electromagnetic wave signals reflected back from targets or strata. The echo data received by the GPR at each detection position is called the A-Scan, and multiple consecutive A-scans on the same detection line can form a B-Scan. B-Scan data are processed in a series of ways to display the subsurface vertical profile in grayscale or pseudo-color images. Figure 2 is the schematic diagram of the GPR detection principle. The underground propagation time of an electromagnetic pulse is shown in Equations (1) and (2):

(1) $$t=\frac{\sqrt{4z^{2}+x^{2}}}{v}$$

(2) $$v=\frac{c}{\sqrt{\varepsilon_{r}}}$$

where *z* is the depth of the target, *x* is the distance between the transmitting antenna and the receiving antenna, *v* is the propagation speed of the electromagnetic wave in the medium, *c* is the speed of light, and *ε_r_* is the relative dielectric constant of the medium.

### 2.3. GPR Image Preprocessing

Due to the interference of complex underground media, external electromagnetic waves, and other factors, the signals received by the GPR during the actual detection process contain a significant amount of noise, which often obscures part of the target echo characteristics. Therefore, it is necessary to preprocess the GPR images to remove these noises to the greatest extent possible, improve the signal-to-noise ratio, and retain effective target reflection information. The main preprocessing methods used in this study were direct wave removal and wavelet denoising.

#### 2.3.1. Removal of Direct Wave

In the echo signals GPR receives, the reflected waves of the ground and the directly coupled waves between the transmitting and receiving antenna are called direct waves. They are prominent in B-Scan images and significantly impact the recognition and extraction of target reflection information. If the direct wave is not processed effectively, the accuracy of the subsequent 3D reconstruction will be seriously reduced.

Since the direct wave data are isochronal and stable, the direct wave data of different measurement lines at the same depth are almost identical. Therefore, the direct wave data can be removed by subtracting the average value of each row of data to enhance the effective reflection data. The expression is shown in Equation (3):

(3) $${\hat{u}}_{k}(t)=u_{k}(t)-\frac{1}{N}\sum_{k=1}^{N}u_{k}(t)$$

where ${\hat{u}}_{k}$(t) is the data of measurement line *k* after removing the direct wave, $u_{k}(t)$ is the raw data of measurement line *k*, and *N* is the number of measurement lines.

#### 2.3.2. Wavelet Denoising

A wavelet transform (WT) is a transformation analysis method that was developed based on the Short-Time Fourier Transform (STFT) [35,36]. The echo signal collected by GPR mainly comprises the target signal and interference noise, which can be expressed as Equation (4):

(4) w(k)=s(k)+u(k)

Equation (5) can be obtained using the above equation’s discrete wavelet transform:

(5) $$w_{j,k}=s_{j,k}+u_{j,k}$$

where *w_j_*_,*k*_, *s_j_*_,*k*_, and *u_j_*_,*k*_ represent the wavelet coefficients corresponding to the echo signal *w*(*k*), target echo signal *s*(*k*), and noise signal *u*(*k*), respectively.

Wavelet denoising involves decomposing the measured signal at different scales by using a wavelet transformation. After the denoising is processed in the wavelet domain, the original signal after denoising is obtained by an inverse transform [37,38]. In this way, the signal interference caused by external conditions can be fully reduced, and then the phase and amplitude transients of the signal can be effectively detected.

**Figure 3:**
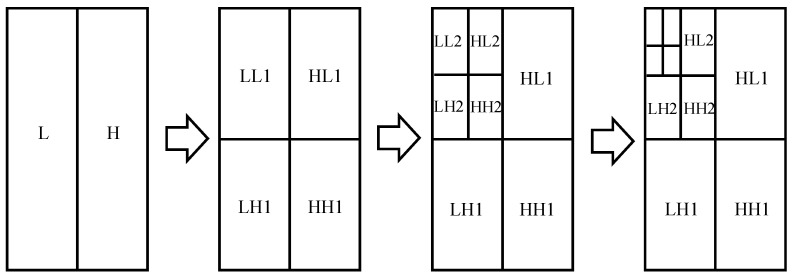
Three-level wavelet decomposition.

The main steps of wavelet denoising are as follows:

(1) Wavelet decomposition: The three-level wavelet decomposition is shown in Figure 3 [39]. Each decomposition will produce a low-frequency (LL) sub-image, horizontal high-frequency (HL) sub-image, vertical high-frequency (LH) sub-image, and high-frequency (HH) sub-image.
(2) Threshold processing: Since most noise signals are in the high-frequency region, only HL, LH, and HH sub-images are processed. Furthermore, the optimized adaptive median-filtering method [40,41] is adopted for denoising to obtain the wavelet coefficients ${\hat{w}}_{j.k}$ after processing.
(3) Wavelet reconstruction: the inverse wavelet transform is used to reconstruct the processed wavelet coefficients ${\hat{w}}_{j.k}$, and the target echo signal $\hat{s}(k)$ after noise removal is attained.

**Figure 4:**
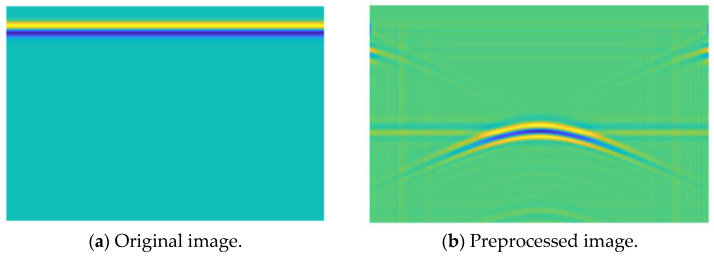
GPR image of the underground cavity model.

Figure 4 shows the radar image of the underground cavity model obtained by gprMax 3.0 software, where (a) is the original image and (b) is the preprocessed image. It can be seen that the effective reflected data in the image were almost covered before preprocessing due to the direct wave being too prominent. After preprocessing, the signal-to-noise ratio of the image was significantly improved, and the target signal could be observed.

### 2.4. BP Imaging Algorithm

Previous studies on GPR imaging algorithms mainly focused on the back-projection (BP) algorithm, the Kirchhoff Migration algorithm, the F-K Migration algorithm, the Diffraction Tomography (DT) algorithm, the Range Migration (RM) algorithm, and the Reverse Time Migration (RTM) algorithm, and others [42,43]. Among them, the BP algorithm fully considers the effect of media stratification in the imaging geometry scene. The refraction of electromagnetic waves at the dielectric interface can be compensated for more precisely and adapted to the road structure [43], so it has great application value in road detection [44].

The BP algorithm is derived from Computer Tomography (CT) imaging technology [45], which was initially one of the imaging methods of Synthetic Aperture Radar (SAR). Since the working principle of GPR is the same as that of SAR, the imaging method of GPR can learn from that of SAR [46]. The principle of the BP algorithm is the coherent superposition of the target scattered energy in the time domain of the received signal to obtain high-resolution imaging results [47,48,49].

**Figure 5:**
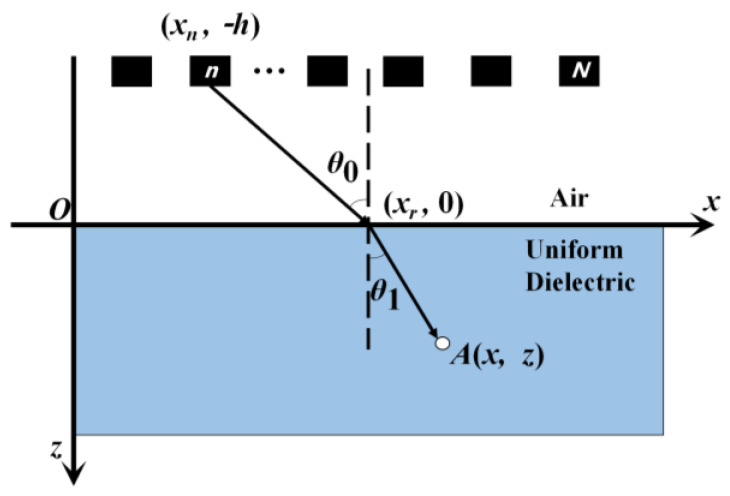
Principle of BP algorithm imaging.

The principle of BP algorithm imaging is shown in Figure 5. The upper layer is air with a permeability of 1 and a relative permittivity of 1, while the lower layer is a uniform dielectric with a permeability of *μ*_1_ and a relative dielectric constant of *ε*_1_. Radar antennas send and receive signals at *N* positions. For the nth antenna, its coordinate is set as (*x_n_*,−*h*), and its electromagnetic wave is refracted at *x_r_*. A random point *A*(*x*, *z*) in the medium is an example for analysis. According to Snell’s law and geometric relations, Equations (6)–(8) can be obtained.

(6) $$\frac{\sin\theta_{0}}{\sin\theta_{1}}=\sqrt{\varepsilon_{1}}$$

(7) $$\sin\theta_{0}=\frac{x_{r}-x_{n}}{\sqrt{h^{2}+{\left(x_{r}-x_{n}\right)}^{2}}}$$

(8) $$\sin\theta_{1}=\frac{x-x_{r}}{\sqrt{z^{2}+{\left(x-x_{r}\right)}^{2}}}$$

where $\theta_{0}$ and $\theta_{1}$ are the incidence angle and refraction angle, respectively. Equation (9) can be obtained by combining Equations (6)–(8):

(9) $${\left(x_{r}-x_{n}\right)}^{2}[z^{2}+{\left(x-x_{r}\right)}^{2}]=\varepsilon_{1}{\left(x-x_{r}\right)}^{2}[h^{2}+{\left(x_{r}-x_{n}\right)}^{2}]$$

According to this equation, the position of the refraction point *x_r_* can be calculated, and then the round-trip propagation time *T_A, n_* of the electromagnetic wave emitted by the antenna at point *A* can be obtained, as shown in Equation (10):

(10) $$T_{A,n}=\frac{2}{c}(\sqrt{h^{2}+{\left(x_{r}-x_{n}\right)}^{2}}+\sqrt{\varepsilon_{1}}\sqrt{z^{2}+{\left(x-x_{r}\right)}^{2}})$$

where *c* is the speed of light.

The amplitude of the nth antenna at point *A* can be obtained by combining *T_A__, n_* and the GPR detection data, which is shown in Equation (11):

(11) $$U_{A,n}={\left.I_{n}(t)\right|}_{t=T_{A,n}}$$

where *I_n_*(*t*) indicates the A-Scan data at the nth antenna position at time *t*.

**Figure 6:**
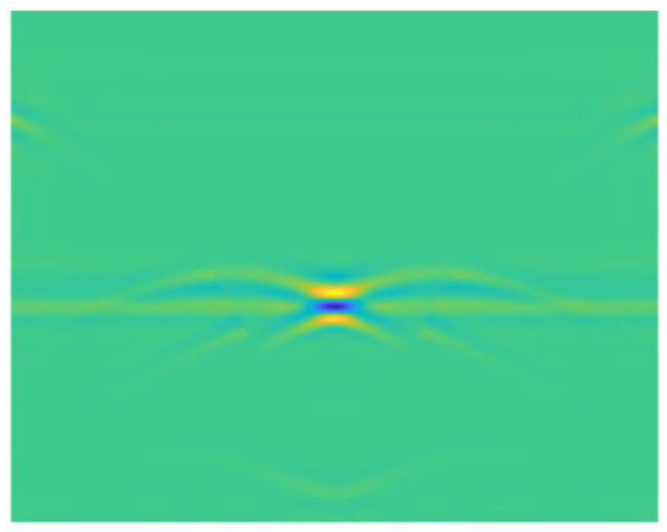
BP imaging.

The final response amplitude of imaging point *A* can be obtained by summing the amplitude of point *A* with the antenna at each position on the measuring line. The above steps are repeated for each point throughout the imaging area to attain the final image result. Taking Figure 4b as an example, the effect of BP imaging is shown in Figure 6.

### 2.5. Improved K-Means Clustering Algorithm

In the process of 3D reconstruction, a threshold should be set to separate and extract the target and background data from the GPR detection data. At present, the setting of this threshold is highly subjective in most studies. Dong Zhou et al. [50] proposed a threshold calculation method based on attribute analysis and K-means clustering analysis, which was successfully applied to the 3D visualization process of tunnel caves and effectively solved the problem of over-reliance on the empirical judgment of researchers in the selection of amplitude threshold in traditional methods.

The K-means clustering algorithm is an unsupervised learning method. Its basic principle is that for a given data set, the data set is divided into *k* clusters according to the Euclidean distance between data points in the sample until the distance between the data in the same cluster are small enough and the distance between clusters is considerable enough [51,52]. Suppose there is a data set *M* = {*x*_1_, *x*_2_, …, *x_m_*} [53], which needs to be clustered into *k* clusters; then, the calculation formula of the sum of the squares of the errors is shown in Equation (12):

(12) $$E=\sum_{i=1}^{k}\sum_{x\in C_{i}}{\left\|x-\mu_{i}\right\|}^{2}$$

Equation (13) shows the corresponding cluster’s mean vector formula:

(13) $$\mu_{i}=\frac{1}{\left|C_{i}\right|}\sum_{x\in C_{i}}x$$

where *k* is the number of clusters, *C_i_* is the cluster, *μ_i_* is the centroid, and *x* is the data point.

**Figure 7:**
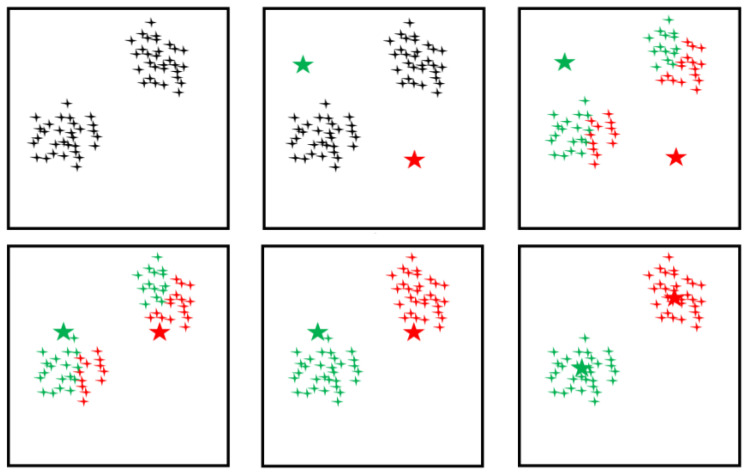
Basic flow of K-means clustering algorithm.

The basic flow of the K-means clustering algorithm is shown in Figure 7. First, *k* samples are selected from data set *M* as the initial clustering center of *k* clusters. According to the minimum Euclidean distance between each data point and the initial clustering center, the data set is divided into *k* clusters. Then, the clustering centers of each cluster are recalculated. The final clustering result can be obtained by repeating the above steps until the *k* clustering centers do not change.

The K-means clustering algorithm has a good classification effect, fast convergence speed, and uncomplicated implementation process, so it is widely used. However, this algorithm is sensitive to noise and abnormal values, and the selection of the initial clustering center will also affect the clustering results of K-means [54]. Aiming at this defect, in recent years, many studies improved the traditional K-means algorithm [55,56,57,58] and achieved good results. Among them, Zhu et al. [58] combined the K-means algorithm with the Agglomerative Hierarchical Clustering (AHC) algorithm and proposed the K-means-AHC algorithm. The AHC algorithm is a hierarchical clustering algorithm with good stability and will not be affected by the cluster number and initial clustering center, but has a high complexity and slow computing speed [59]. Therefore, the K-means-AHC algorithm combines the advantages of these two algorithms. First, the data set is divided into several initial class clusters using the K-means algorithm processing method. Second, based on the initial class cluster formed, the AHC algorithm is adopted to merge the smaller initial class cluster into a larger class cluster that meets the requirements. This method not only preserves the fast operation time but also avoids the acute problem of the traditional K-means algorithm regarding selecting the initial clustering center.

## 3. Results and Discussion

In this section, the 3D reconstruction method of road structural defects proposed above was verified and evaluated by combining the GPR profile data simulated by gprMax software and measured by laboratory tests.

### 3.1. Simulated GPR Data

gprMax is an open-source simulation software developed by Antonis Giannopoulos and others based on the Finite-Difference Time Domain (FDTD) and Perfectly Matched Layer (PML) absorbing boundary [60,61]. Its primary function is to simulate the propagation of GPR electromagnetic waves. Due to its advantages of solid professionalism, high computational efficiency, convenient parameter setting, and realistic simulation results, gprMax is very suitable for simulating GPR for road structure detection.

In this study, defects of different types and sizes located in the road interior were taken as the primary research objects, and the basic parameters of the gprMax model are shown in Table 1. Four media layers were set in the model, and the parameters of each layer are shown in Table 2.

**Figure 8:**
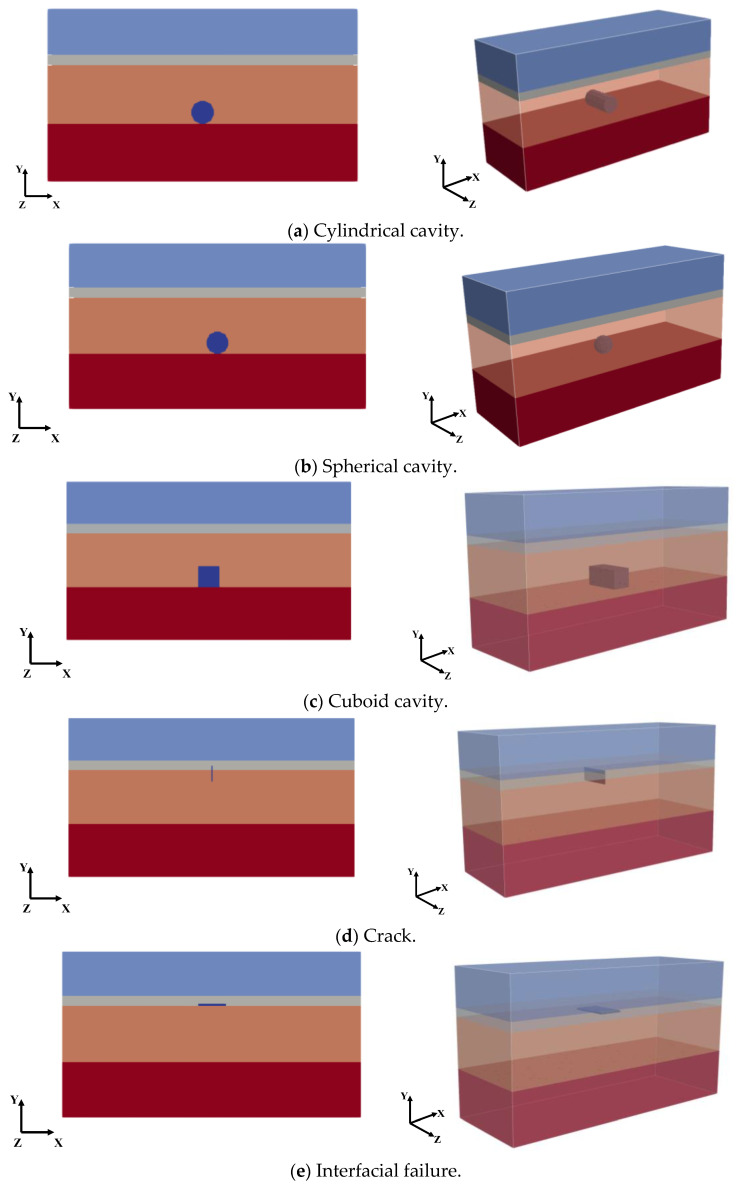
Defect model.

We set the different types and sizes of defects inside the model, as shown in Table 3. The material parameters of the defects were set to air. Taking models No. 4, No. 8, No. 12, No. 24, and No. 30 as examples, the relative positions of the defects are shown in Figure 8, where (a) represents a cylindrical cavity defect, (b) represents a spherical cavity defect, (c) represents a cuboid cavity defect, (d) represents a crack defect, and (e) represents an interfacial failure defect.

**Figure 9:**
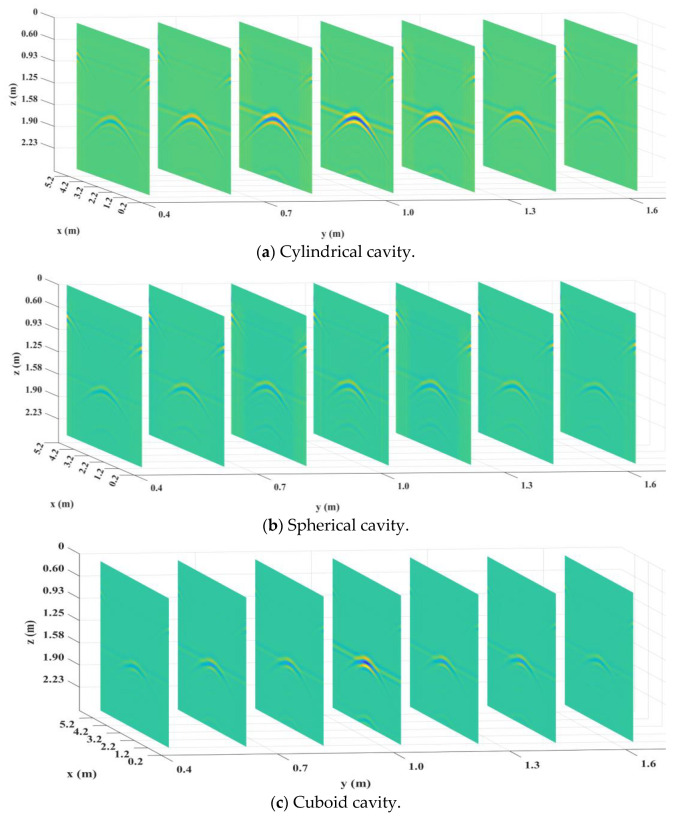

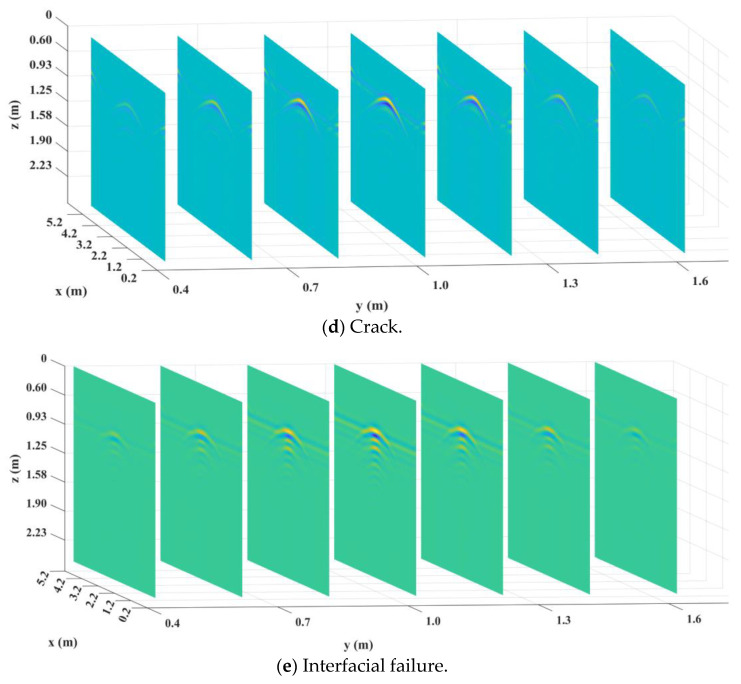
B-Scan images.

After the models were established, an XZ section was selected along the Y-axis every 20 cm, and the transmitting antenna and receiving antenna were placed above each section for the GPR detection. After the gprMax processing, a group of B-Scan images were obtained, which were preprocessed as shown in Figure 9.

**Figure 10:**
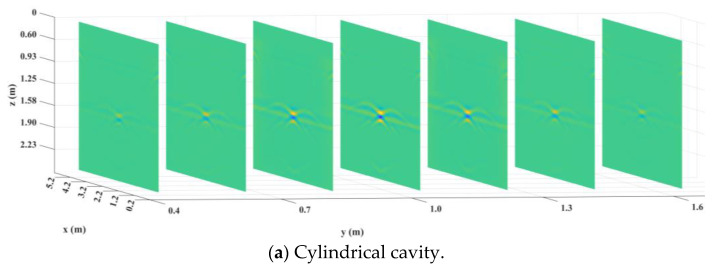

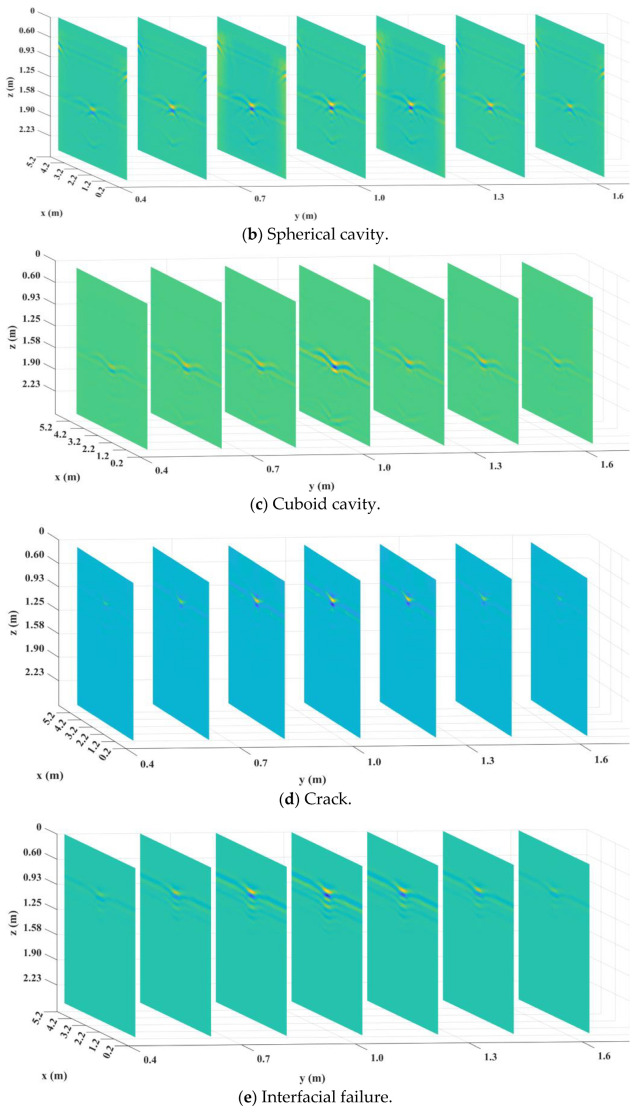
Results of BP imaging.

The above images were processed by BP imaging, and the processing results are shown in Figure 10. It can be seen that after the pretreatment and BP image processing, the concentrated position of the reflected signal in the image was consistent with the actual position of the defect.

**Figure 11:**
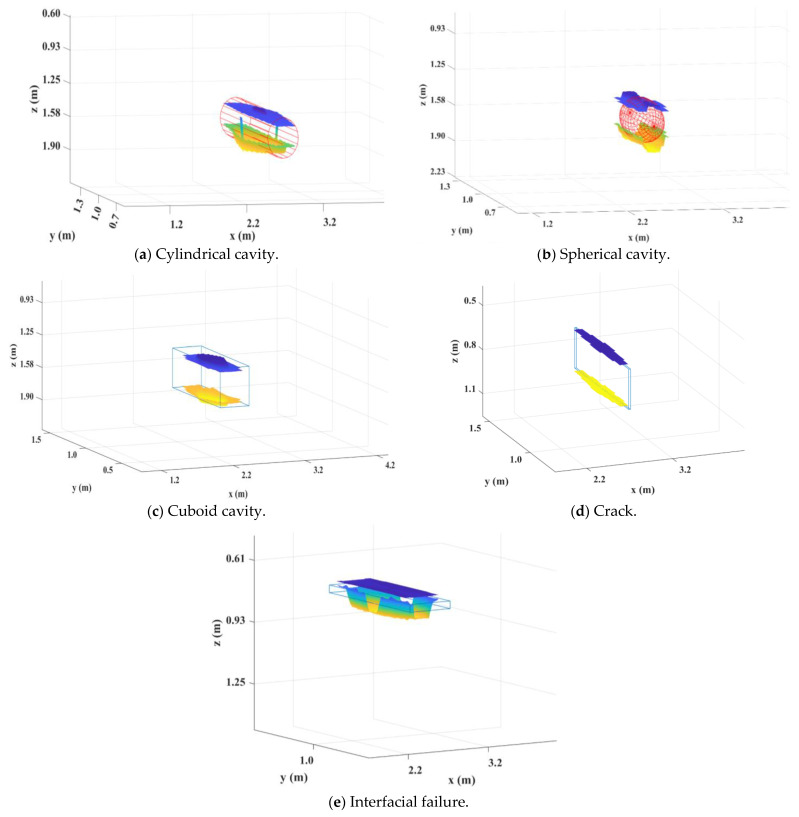
Results of 3D reconstruction.

Next, the radar data of each section after the preprocessing and BP imaging processing were interpolated in MATLAB to construct a 3D data set. Then, the K-means-AHC algorithm was used to calculate the threshold. All data were divided into background data and effective reflection data according to the threshold, and all background data were set to 0. Finally, the upper and lower surfaces of the effective reflection data were extracted and imaged in MATLAB, as shown in Figure 11. The red or blue part of the diagram represents the actual defect.

The central position of the reconstructed defects is shown in Table 4. Combined with the image, the size and position of the reconstructed defect were consistent with the actual size and position of the defect in the model.

In order to further evaluate the accuracy of the reconstruction, the concept of Intersection Over Union (*IoU*) in object detection is introduced in this paper, namely, the ratio of the intersection and union between the actual volume and the reconstructed volume of the defect, as shown in Equation (14):

(14) $$IoU=\frac{V_{T}\cap V_{R}}{V_{T}\cup V_{R}}$$

where *V_T_* is the actual volume of the defect in the model, while *V_R_* is the volume of the reconstructed defect. If they overlap perfectly, then *IoU* is 1. Generally speaking, if the *IoU* is greater than or equal to 0.5, the results can be considered to be relatively accurate [62]. Table 5 shows the *IoU*s of the above six simulated defects.

According to the above data, it can be seen that the method proposed in this paper could realize the 3D reconstruction of road structural defects, such as cavities, cracks, and interfacial failures, accurately, and the *IoU* could reach about 0.6. The larger the size of the diseases and the closer they were to the road surface, the higher the accuracy of the reconstruction results.

### 3.2. Measured GPR Data

In order to further verify the application of the above methods in practical engineering, several sets of laboratory tests were carried out in this study. The IDS-RIS GPR produced in Italy was used in the test. The antenna was selected as the ground-coupled TR-HF antenna and the frequency was selected as 1.6 GHz, which was chosen to provide higher resolution.

**Figure 12:**
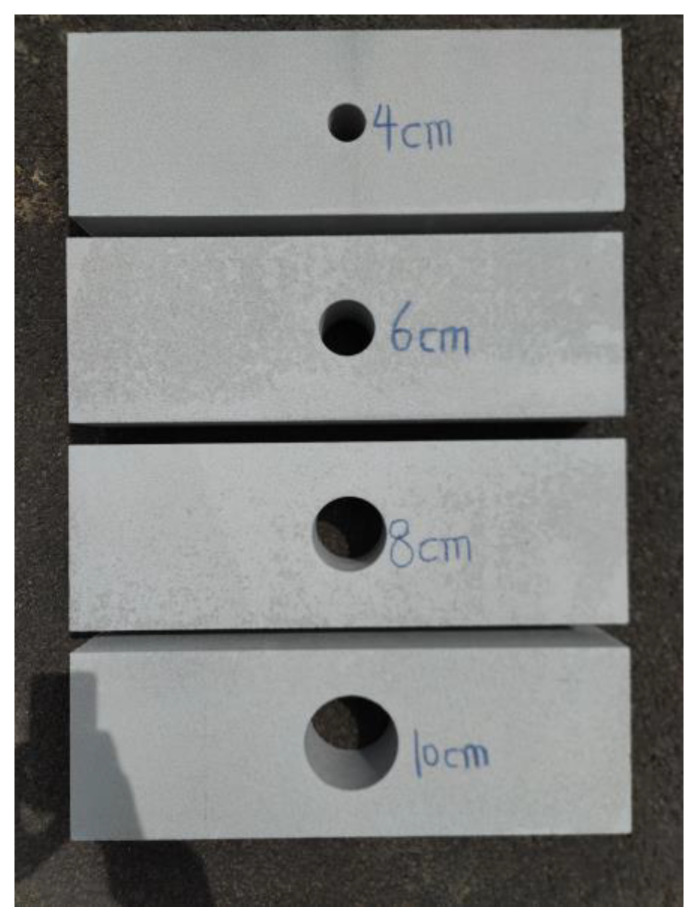
Laboratory test model.

**Figure 13:**
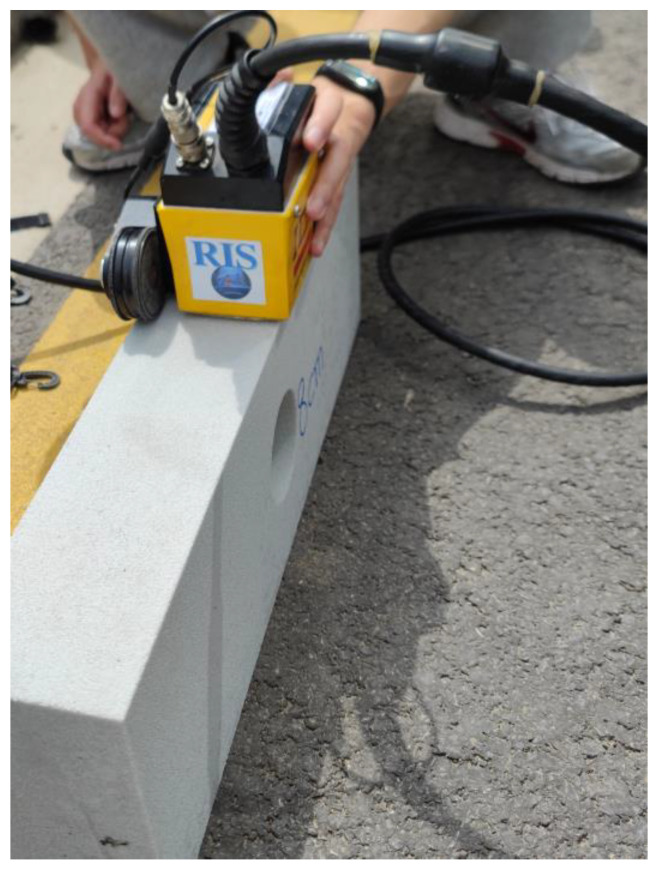
IDS-RIS GPR.

Concrete specimens were used to simulate the base. The size of the specimens was 60 cm × 10 cm × 20 cm. Cylindrical cavities with diameters of 4 cm, 6 cm, 8 cm, and 10 cm were present. GPR detection was performed on each of these specimens. The test site is shown in Figure 12 and Figure 13.

**Figure 14:**
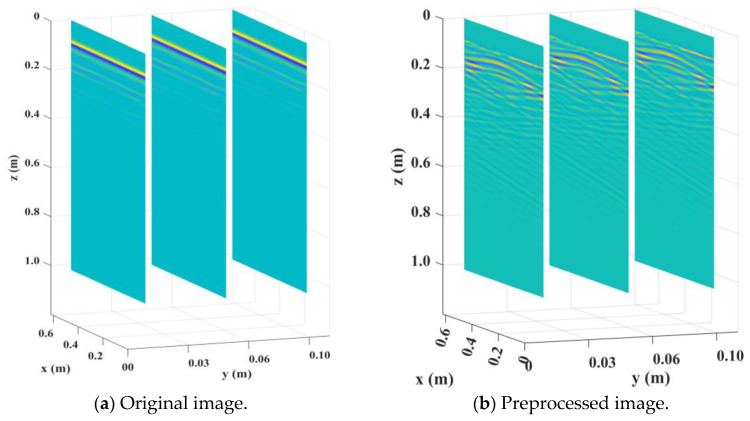

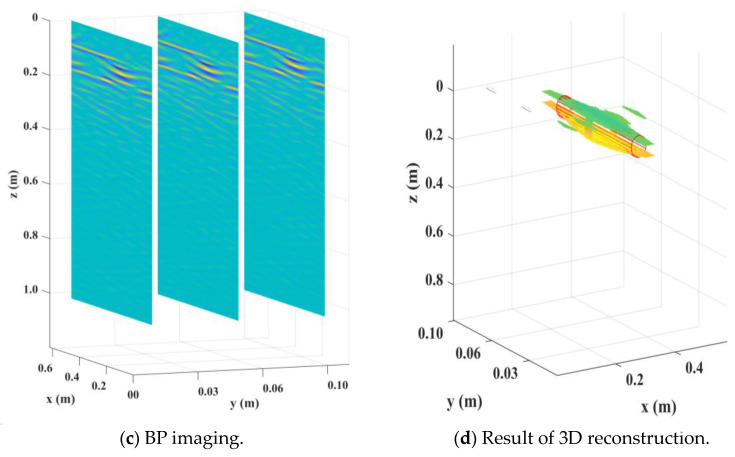
B-Scan images and processing results of laboratory test.

Taking the defect model with a diameter of 6 cm as an example, the detected original B-Scan image and the processing results of the preprocessing and BP imaging are shown in Figure 14, where (a) is the original image, (b) is the preprocessing image, (c) is the image processed by the BP algorithm, and (d) is the result of the three-dimensional reconstruction.

The central position and *IoU* of the reconstructed defects are shown in Table 6. Compared with the results of the software simulation, the accuracy of the 3D reconstruction was somewhat reduced in the actual measurement process due to the more complex media composition and more significant interference from environmental noise. However, it was still within an acceptable range.

**Figure 15:**
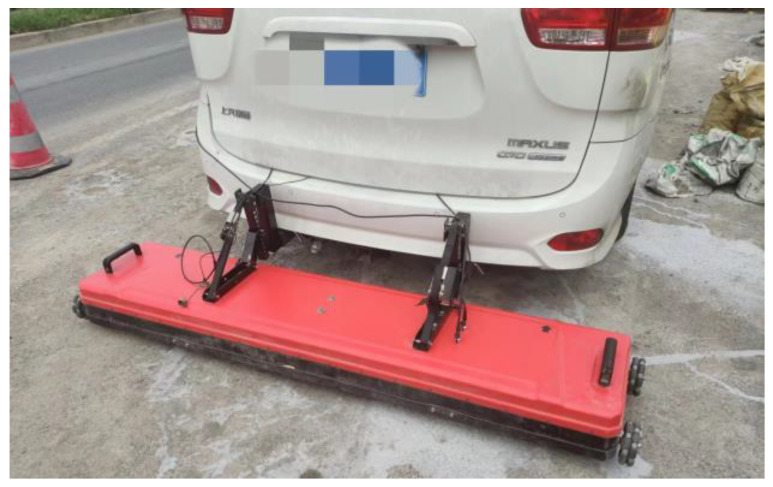
Radar used in detection.

Then, the above method was applied to the actual road for verification. The test road was an asphalt road located in Jinhua, Zhejiang, China, and the length of the section tested was about 1.07 km. The radar used for detection is shown in Figure 15, which had a detection depth of 8 m and a detection speed of 60 km/h.

According to the field test results, the main structural defects of this road included cavities, cracks, and looseness.

**Figure 16:**
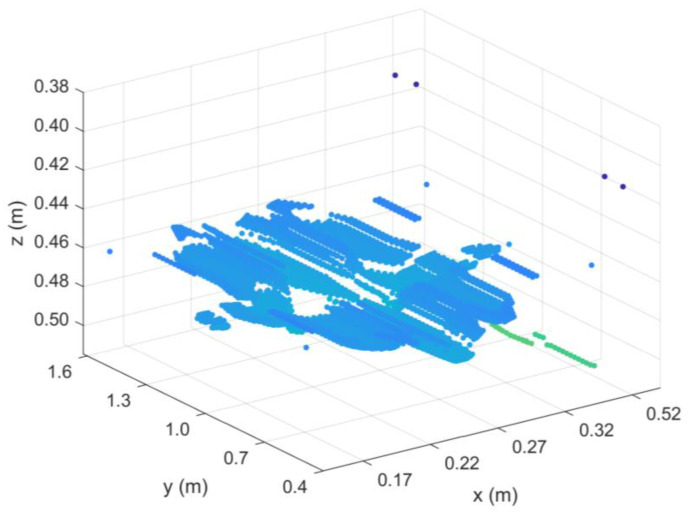
Result of 3D reconstruction on the actual road.

According to the 3D reconstruction algorithm proposed in this paper and relevant municipal data, the location and size of the structural defects in this road were further judged and analyzed. Taking the second lane on the right as an example, some results obtained from the detection and calculation are summarized in Table 7. An example of the 3D reconstruction results is shown in Figure 16.

**Figure 17:**
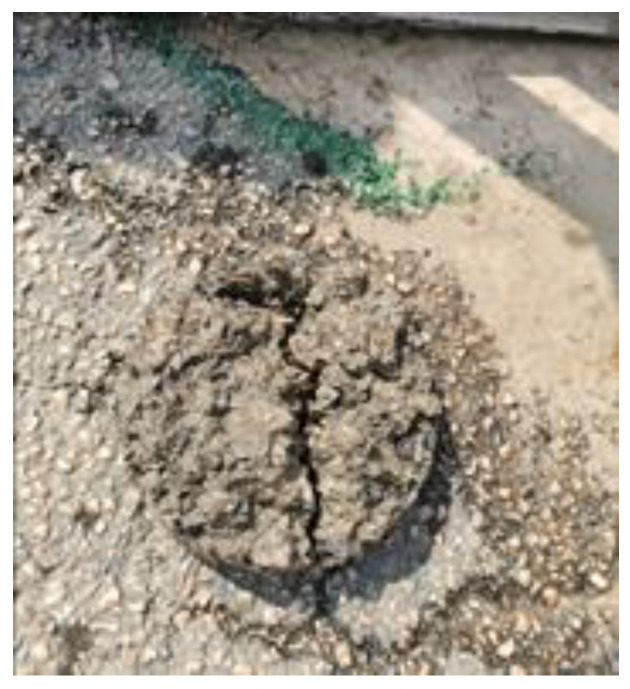
Core sample.

Considering the future traffic demand of the road, we drilled the core of only part of the area, as shown in Figure 17. Through these limited core samples, we found that due to the complexity of the actual road conditions, compared with the real defects, the algorithm found it more difficult to recover the shape of the defects, but the position and volume of the defects calculated by the algorithm were basically consistent with the actual situation, and the *IoU* could reach above 0.5.

## 4. Conclusions

This paper proposes a 3D reconstruction method for road structural defects by combining the BP imaging algorithm with GPR detection data. The method was validated using both gprMax simulations and laboratory test models. The main conclusions were as follows:

(1) Through the gprMax simulation images, it was observed that the hyperbolic reflection feature was the most prominent at the central position of the defect and gradually weakened toward the edges.
(2) The BP algorithm effectively facilitated the accurate resetting of radar waves, which ensured that the reflected signal was concentrated at the actual location of the defect.
(3) For the gprMax model, the method proposed in this paper could accurately realize the 3D reconstruction of the road structural defects, such as cavities, cracks, and interfacial failures, and achieved an *IoU* of approximately 0.6. Additionally, the larger the size of the defects and the closer they were to the road surface, the higher the accuracy of the reconstruction results. For the laboratory test model, the accuracy of the 3D reconstruction was reduced due to the complex media composition and significant interference from environmental noise. Nevertheless, the average *IoU* was around 0.52, which is still acceptable.
(4) Combined with the actual road-drilling coring results, it could be verified that the algorithm could detect and reconstruct the road structural defects more accurately.

The results of this work hold significant implications for the detection and maintenance of road structural defects. However, a notable limitation of this study was the simplification of defects into regular geometric shapes. Additionally, in the gprMax model, the materials of each structural layer were assumed to be uniform, with no consideration of other substances. The accurate detection of road structural defects continues to pose challenges. Future research should aim to acquire more measured data and develop more precise models to enhance the reliability of defect detection.

## Figures and Tables

**Table 1:** Basic parameters of the gprmax model.

Parameter	Value
The model size (m)	5.4 × 2.0 × 3.0
The grid step (m)	0.02 × 0.02 × 0.02
The width of PML (m)	0.2
Time window (ns)	44
Center frequency (MHz)	300
Type of excitation source	Ricker
The initial coordinate of transmitting antenna (m)	(0.2, 1.0, 2.8)
The initial coordinate of transmitting antenna (m)	(0.2, 1.0, 2.8)
The moving step of the antenna (m)	0.02
The number of measured lines	251

**Table 2:** Parameters of the medium at each layer of the model.

Structural Layer	Relative Permittivity	Electrical Conductivity (S/m)
Air	1	0
Surface	6	0.005
Base	8	0.005
Subgrade	12	0.1

**Table 3:** Type, size, and location of defects in the model.

Model No.	Type of Defect	Location	Size (m)
1	Cylindrical cavity	Base	Length: 0.8 m, diameter: 0.1 m
2	Cylindrical cavity	Base	Length: 0.8 m, diameter: 0.15 m
3	Cylindrical cavity	Base	Length: 0.8 m, diameter: 0.2 m
4	Cylindrical cavity	Base	Length: 0.8 m, diameter: 0.25 m
5	Spherical cavity	Base	Diameter: 0.1 m
6	Spherical cavity	Base	Diameter: 0.15 m
7	Spherical cavity	Base	Diameter: 0.2 m
8	Spherical cavity	Base	Diameter: 0.25 m
9	Cuboid cavity	Base	0.8 m × 0.1 m × 0.1 m
10	Cuboid cavity	Base	0.8 m × 0.15 m × 0.15 m
11	Cuboid cavity	Base	0.8 m × 0.2 m × 0.2 m
12	Cuboid cavity	Base	0.8 m × 0.25 m × 0.25 m
13	Crack	Surface	Width: 5 mm, length: 0.1 m
14	Crack	Surface	Width: 10 mm, length: 0.1 m
15	Crack	Surface	Width: 15 mm, length: 0.1 m
16	Crack	Surface	Width: 20 mm, length: 0.1 m
17	Crack	Base	Width: 5 mm, length: 0.3 m
18	Crack	Base	Width: 10 mm, length: 0.3 m
19	Crack	Base	Width: 15 mm, length: 0.3 m
20	Crack	Base	Width: 20 mm, length: 0.3 m
21	Crack	Surface and base	Width: 5 mm, length: 0.3 m
22	Crack	Surface and base	Width: 10 mm, length: 0.3 m
23	Crack	Surface and base	Width: 15 mm, length: 0.3 m
24	Crack	Surface and base	Width: 20 mm, length: 0.3 m
25	Interfacial failure	Surface	0.5 m × 0.8 m × 0.01 m
26	Interfacial failure	Surface	0.5 m × 0.8 m × 0.02 m
27	Interfacial failure	Surface	0.5 m × 0.8 m × 0.04 m
28	Interfacial failure	Base	0.5 m × 0.8 m × 0.01 m
29	Interfacial failure	Base	0.5 m × 0.8 m × 0.02 m
30	Interfacial failure	Base	0.5 m × 0.8 m × 0.04 m

**Table 4:** Central coordinate of reconstructed defects.

Model No.	Calculated Center Coordinate (m)	Actual Center Coordinate (m)
1	(2.693, 1.006, 1.776)	(2.700, 1.000, 1.750)
2	(2.693, 1.006, 1.743)	(2.700, 1.000, 1.725)
3	(2.698, 1.005, 1.716)	(2.700, 1.000, 1.700)
4	(2.698, 1.005, 1.684)	(2.700, 1.000, 1.675)
5	(2.700, 1.009, 1.779)	(2.700, 1.000, 1.750)
6	(2.700, 1.009, 1.749)	(2.700, 1.000, 1.725)
7	(2.700, 1.005, 1.711)	(2.700, 1.000, 1.700)
8	(2.700, 1.005, 1.690)	(2.700, 1.000, 1.675)
9	(2.692, 1.006, 1.776)	(2.700, 1.000, 1.750)
10	(2.692, 1.006, 1.746)	(2.700, 1.000, 1.725)
11	(2.693, 1.005, 1.713)	(2.700, 1.000, 1.700)
12	(2.693, 1.005, 1.697)	(2.700, 1.000, 1.675)
13	(2.695, 1.004, 1.012)	(2.700, 1.000, 0.890)
14	(2.695, 1.004, 1.003)	(2.700, 1.000, 0.890)
15	(2.700, 1.003, 0.981)	(2.700, 1.000, 0.890)
16	(2.700, 1.003, 0.927)	(2.700, 1.000, 0.890)
17	(2.690, 1.008, 1.644)	(2.700, 1.000, 1.490)
18	(2.690, 1.007, 1.593)	(2.700, 1.000, 1.490)
19	(2.699, 1.003, 1.551)	(2.700, 1.000, 1.490)
20	(2.700, 1.003, 1.509)	(2.700, 1.000, 1.490)
21	(2.692, 1.007, 1.210)	(2.700, 1.000, 1.050)
22	(2.692, 1.007, 1.162)	(2.700, 1.000, 1.050)
23	(2.700, 1.005, 1.095)	(2.700, 1.000, 1.050)
24	(2.700, 1.004, 1.083)	(2.700, 1.000, 1.050)
25	(2.703, 1.001, 0.986)	(2.700, 1.000, 0.975)
26	(2.701, 1.000, 0.975)	(2.700, 1.000, 0.970)
27	(2.700, 1.000, 0.964)	(2.700, 1.000, 0.960)
28	(2.705, 1.002, 2.009)	(2.700, 1.000, 1.995)
29	(2.702, 1.001, 2.002)	(2.700, 1.000, 1.990)
30	(2.702, 1.000, 1.987)	(2.700, 1.000, 1.980)

**Table 5:** *IoU* of simulated defects.

**Model No.**	1	2	3	4	Mean
***IoU***	0.650	0.687	0.721	0.735	0.698
**Model No.**	5	6	7	8	Mean
***IoU***	0.624	0.668	0.677	0.697	0.667
**Model No.**	9	10	11	12	Mean
***IoU***	0.674	0.696	0.715	0.729	0.704
**Model No.**	13	14	15	16	Mean
***IoU***	0.564	0.591	0.654	0.705	0.629
**Model No.**	17	18	19	20	Mean
***IoU***	0.534	0.593	0.612	0.628	0.592
**Model No.**	21	22	23	24	Mean
***IoU***	0.563	0.587	0.609	0.644	0.601
**Model No.**	25	26	27	-	Mean
***IoU***	0.636	0.675	0.704	-	0.672
**Model No.**	28	29	30	-	Mean
***IoU***	0.621	0.643	0.685	-	0.650

**Table 6:** Three-dimensional reconstruction results of laboratory test.

Diameter of Cavity (cm)	Calculated Center Coordinate (cm)	Actual Center Coordinate (cm)	*IoU*
4	(30.22, 4.86, 9.32)	(30, 5, 10)	0.505
6	(30.47, 4.75, 9.41)	(30, 5, 10)	0.519
8	(30.43, 4.83, 9.05)	(30, 5, 10)	0.517
10	(30.61, 4.43, 8.93)	(30, 5, 10)	0.521

**Table 7:** Three-dimensional reconstruction results of the second lane on the right.

No.	Defect Type	Initial Position	Termination Position	Initial Depth (m)	Termination Depth (m)	Size (m)
1	Pipeline	K62 + 96.70	K62 + 94.53	0.47	1.21	2.17 × 0.74
2	Looseness	K62 + 76.60	K62 + 74.55	0.47	0.57	2.05 × 0.10
3	Cavity	K62 + 71.66	K62 + 66.56	0.55	0.64	5.10 × 0.09
4	Crack	K62 + 31.99	K62 + 31.08	0.42	0.45	0.91 × 0.03
5	Pipeline	K62 + 12.10	K62 + 10.57	0.45	1.03	1.53 × 0.59
6	Pipeline	K62 + 9.46	K62 + 8.96	0.43	1.19	0.50 × 0.76
9	Cavity	K61 + 990.09	K61 + 988.65	0.48	0.86	1.44 × 0.38
10	Cavity	K61 + 987.62	K61 + 986.82	0.65	0.91	0.80 × 0.26
11	Pipeline	K61 + 977.32	K61 + 976.78	0.10	1.47	0.54 × 1.37
12	Crack	K61 + 951.53	K61 + 949.85	0.60	0.82	1.68 × 0.02
13	Cavity	K61 + 943.29	K61 + 941.57	0.54	0.82	1.72 × 0.27
14	Looseness	K61 + 935.49	K61 + 932.42	0.45	0.95	3.07 × 0.50
15	Cavity	K61 + 930.00	K61 + 925.42	0.43	0.64	4.58 × 0.21
16	Cavity	K61 + 921.64	K61 + 919.06	0.40	0.94	2.58 × 0.54
17	Cavity	K61 + 918.52	K61 + 915.40	0.40	0.59	3.12 × 0.19
18	Looseness	K61 + 873.15	K61 + 870.80	0.43	1.03	2.35 × 0.60
19	Cavity	K61 + 813.11	K61 + 810.35	0.77	0.97	2.76 × 0.19
20	Crack	K61 + 802.86	K61 + 802.83	0.38	1.00	0.03 × 0.61

## Data Availability

Restrictions apply to the availability of these data. The data can be obtained from the corresponding author upon request.
